# Identifying dead regions in the cochlea through the TEN Test

**DOI:** 10.1016/S1808-8694(15)31025-9

**Published:** 2015-10-19

**Authors:** Regina TS Jacob, João Cândido Fernandes, Jair Manfrinato, Maria Cecília M Iório

**Affiliations:** a Doctor on Rehabilitation Science/Human Communication Disorders at the Sao Paulo University Craniofacial Anomalies Rehabilitation Hospital/Bauru Campus (HRAC/USP/Bauru), Speech Therapist of the CEDALVI/HRAC/USP Bauru.; b Lecturer at UNESP/Bauru, Professor of the post-graduate program on Rehabilitation Science/Human Communication Disorders at HRAC/USP/Bauru.; c Doctor on Agricultural Science at UNESP/Botucatu, Head of the Production Engineering Department at UNESP/Bauru.; d Doctor on Human Communication Disorders at the UNIFESP/EPM, Adjunct Professor of the Speech Therapy Course at UNIFESP/EPM. Sao Paulo University Craniofacial Anomalies Rehabilitation Hospital/Bauru Campus.

**Keywords:** Hearing Loss, Hearing Aids, Cochlea, Dead Cochlear Regions

## Abstract

An audiogram is not sufficient to indicate cochlear dead regions.

**Aim:**

To investigate cochlear dead regions in sensorineural hearing loss subjects using the TEN test. Site: CEDALVI/ HRAC-USP-Bauru/Sao Paulo/Brazil, August 2003 to February 2004.

**Study Design:**

A contemporary cross-sectional cohort study. Material and Methods: The TEN test was applied in three groups: G1(5 women with pure-tone thresholds within normal limits); G2(4 women and 5 men with moderate sensorineural flat hearing loss); G3(19 women and 24 men with mild to severe sloping sensorineural hearing loss).

**Results:**

In the G1 group the TEN value required to eliminate the test tone was, on average, close to the absolute threshold for all frequencies. No dead regions were found in the ears tested in group G2. 76 ears were tested in group G3, and six showed no evidence of dead regions in the cochlea.

**Conclusions:**

The TEN test was an effective test to indicate a dead region in the cochlea of subjects with sloping sensorineural hearing loss. There is evidence that pure-tone detection is different for subjects with high frequency sensorineural hearing loss and flat hearing loss; we observed a significant difference between the masked threshold and the absolute threshold only in sloping hearing loss and not for flat hearing loss.

## INTRODUCTION

### Cochlear pathophysiology and dead regions in the cochlea

The cochlear region where internal ciliated cells (ICC) are injured, inactive or absent, and the neurons that innervate this region are inactive or even degenerate, has been named dead region of the cochlea, or dead region.^1^, ^2^, ^3^

Although the dead region concept was elaborated many years ago by Gravendeel and Plomp,^4^ it generated little medical interest until Moore et al., in recent papers on this topic, brought the term back to use. This author describes dead regions based on the frequency of injured ICCs and/or neurons belonging to the dead region in question.^3^, ^5^, ^6^

Audiogram configurations have also been considered as evidence or dead regions, such as sloping audiograms, where thresholds worsen abruptly with increasing pitch (over 50 dB/octave) at high frequencies. Hearing losses of 40-50 dB at low frequencies and hearing close to normal in medium and high frequencies may suggest dead regions at low frequencies. This may also be the case when there is hearing loss over 50 dB at low frequencies with improved thresholds at higher frequencies. A U-shaped audiogram, with better hearing at low and high frequencies and a dead region at middle frequencies, is rare and generally does not interfere significantly with speech recognition. Residual hearing allows good recognition, meaning that some of these cases do not require hearing aids (individual sound amplification devices).^5^

Huss et al. investigated pure tone perception in subjects with and without dead regions diagnosed by psychophysical tuning curves and the threshold-equalizing-noise (TEN) test, in which participants were asked to score the sharpness of a pure tone on a scale from sharp (1) to noisy (7). The authors concluded that the pure tone subjective impression of sharpness is not a consistent indicator of dead regions in the cochlea, as higher scores were seen not only in ears with dead regions but also in normal ears at similar frequencies and at higher thresholds.^7^

It is clear that patient reports or the audiogram by themselves are not sufficient to establish or exclude the presence and extension of dead regions. Thus, masking has been used in some studies to investigate this condition in greater detail.

### Assessment of dead regions in the cochlea

The investigation of dead regions uses ipsilateral masking, in which the test signal and the masking noise are presented to the same ear. The idea is to raise the threshold of non-tested frequencies so that they do not respond to excitation diffusion of the signal that is being tested at a specific frequency.

Moore et al. developed the Threshold-Equalizing-Noise test to investigate the presence of dead regions. Although masking is defined as a procedure used in audiological evaluation, and not a test in itself, TEN is considered a test by its authors. It is based on the detection of pure tones presented simultaneously with a wide band noise (TEN) which produces practically the same level of masking (measured in dBNPS) throughout audiogram frequencies (250 Hz to 10,000 Hz) in normal hearing or hearing-impaired individuals with no dead regions.^3^, ^5^

In Brazil Eguti investigated the efficiency of the masking technique using white noise to identify dead regions in the cochlea of 32 adult individuals that presented acquired sensorineural or mixed hearing loss and a sloping audiometric configuration. Moore's^5^ criteria and masking test results using white noise showed a strong correlation (kappa index of agreement = 96.1%). The author concluded that the masking technique using white noise is a reliable and simple test for routine use in a clinical setting to test for dead regions in the cochlea for cases of acquired sensorineural hearing loss in a sloping configuration.^8^

The diagnosis of dead regions in the cochlea is important in medical practice, as studies have shown that its presence limits the use of hearing aids, since these regions respond minimally or not at all to sound amplification.^5^, ^6^, ^9^

## OBJECTIVE

Our aim was to use the TEN test to investigate the presence of dead regions in the cochlea in subjects with sensorineural hearing loss seen at the Center for Audition, Vision and Language Disorders (CEDALVI) of the Sao Paulo University Craniofacial Anomalies Rehabilitation Hospital/Bauru Campus (HRAC-USP-Bauru).

## MATERIAL AND METHODS

This study was undertaken at the CEDALVI/HRAC/USP-Bauru between August 2003 and February 2004.

### CASES

 

### Profile

According to the routine at CEDALVI, patients are monitored by a multidisciplinary audiology team. Participants were assessed by an ENT specialist who did otoscopy and decided whether conditions were appropriate for the TEN test, namely intact tympanic membranes and no external and middle ear compromise.

Cases for this study are shown on Table 1.

**Table 1:** Number of subjects, ears and audiograms for groups G1, G2 and G3.

Groups	Number of subjects	Number of ears	Audiogram
G1	5	10	normal (≤15dBNA)
G2	9	15	mild flat sensorineural hearing loss
G3	43	76	moderate to severe sloping sensorineural hearing loss

TOTAL	57	101	

### Selection criteria

The selection of subjects was based on the following inclusion criteria:

a) G1: pure tone air conduction thresholds within normal limits (≤15dBNA);
b) G2: mild flat configuration sensorineural hearing loss (Davis and Silverman);^10^
c) G3: moderate to severe sloping sensorineural hearing loss.

### General ethical aspects

Participants signed a free informed consent form approved by the Research Ethics Committee of the HRAC-USP, number 263/2004-UEP-CEP, agreeing to take part in the study and to allow publication of collected data.

## METHODS

### Instrument and Procedure

a) TEN biological calibration
  We used the Veniar^11^ biological calibration technique and the procedure consisted of simultaneously and ipsilaterally (through the same earphone) presenting a pure tone at an initial intensity of 30 dBNPS (minimal audiometer setting) and noise (TEN), which was increased in 5 dB steps until it masked the test stimulus.
b) TEN test
  The TEN test was applied using a two-channel audiometer, PAC 2000 model (Acustica Orlandi) with a TDH-39 P supra-aural earphone and a Sony Discman digital MEGA BASS model, in a soundproofed cabin. Moore et al. (2000) recorded the test on a CD with noise (TEN) on one channel and a digitally generated test signal (pure tone) recorded on another CD channel. This was done to simplify and increase the clinical availability of the TEN test.

The following test procedures were undertaken:

(1) the CD player output plug was connected to the audiometer right and left input socket (usually used for tape input). The first CD track is a calibrating tone, which was played to adjust the audiometer right and left channel VU meters at -5 dB, or 5 dB below zero; this was done to calibrate the signal-to-noise ratio, or the intensity difference between the pure tone and noise. For the remaining tracks the noise intensity level in the right channel was read as 10 dB better than the indicated level on the dial, and the left channel pure tone was read as 10 dB worse. We could then adjust the audiometer controls for the desired intensities for pure tones and noise. Thus, if the pure tone and noise intensities were 50 and 40dBNPS, the dial was adjusted to 40 and 50 dB, respectively.
(2) Air conduction absolute thresholds (ATs) and masked thresholds (MTs) were obtained by manual audiometry, according to Carhart and Jerger,^7^ with stimuli controlled on the audiometer to which the CD was attached. ATs were assessed as dBNPS at frequencies of 250, 500, 1,000, 2,000, 3,000, 4,000, 6,000, and 8,000 Hz, using test pure tones recorded on the CD left channel. This procedure was applied separately to each ear; there was no need to use contralateral masking, as in no case the opposite ear had a sufficient threshold to respond for the tested ear.
(3) MTs with noise (TEN) were assessed at the same frequencies, according to the audiometric configuration, with both channels operating. The TEN intensity depended on the AT by frequency in each patient, and was 10 dB better than the AT, that is, if the AT was 50 dB, the TEN was adjusted to 40 dB on the dial.
(4) If the MT was only 5 dB worse than the AT, the test was repeated with higher masking noise intensity.

### Data analysis

 

### Criteria for result analysis

a) Biological calibration of the TEN
  The minimal effective masking level considered was the amount of noise (TEN) that was needed to eliminate the test tone (30 dBNPS) for each frequency.
b) TEN test
  According to Moore et al.'s^3^ criteria, dead regions in the cochlea were suggested when, at a specific frequency, the MT was at least worse by 10 dB than the AT and 10 dB worse than the masking noise (TEN). Absence of dead regions was indicated if the MT was equal to the AT. The result was considered as inconclusive if the difference between the AT and the MT was 5 dB, or if intensity on the audiometer was not sufficient to assess the MT.
c) Statistical method
  The following statistical tests were used: t test (test to compare two paired samples, or the t-student test), and Spearman's rank correlation coefficient. The null hypothesis rejection value (Ho) was 5%.

## RESULTS

### Study of the variation of absolute thresholds using the TEN

Table 2 shows the results for absolute thresholds and threshold values obtained using TEN in group G1.

**Table 2:** TEN values (in dBNPS) needed to eliminate the test tone (AT) in 10 group 1 ears (G1).

Ear	FREQUENCY - HZ															
	250		500		1000		2000		3000		4000		6000		8000	
	AT	TEN	AT	TEN	AT	TEN	AT	TEN	AT	TEN	AT	TEN	AT	TEN	AT	TEN
1	30	30	30	35	30	35	30	30	30	35	30	30	30	30	30	30
2	30	30	30	30	30	30	30	35	30	35	30	30	30	30	30	30
3	30	30	30	30	30	30	30	30	30	35	30	30	30	30	30	25
4	30	30	30	30	30	25	30	30	30	30	30	30	30	30	30	30
5	30	30	30	30	30	35	30	35	30	30	30	30	30	35	30	30
6	30	30	30	30	30	30	30	30	30	35	30	35	30	25	30	30
7	30	30	30	35	30	35	30	30	30	30	30	35	30	30	30	30
8	30	30	30	30	30	35	30	35	30	30	30	35	30	30	30	30
9	30	30	30	35	30	35	30	30	30	30	30	30	30	35	30	35
10	30	30	30	35	30	35	30	30	30	35	30	30	30	35	30	35

Average		32,00		32,50		31,50		31,67		32,50		31,50		31,00		30,50

S.D.		2,58		3,54		2,42		2,50		2,64		2,42		3,16		2,84

**Legend:** AT absolute threshold TEN masking noise S.D. standard deviation

As seen on Table 2, the TEN value needed to eliminate the test tone (AT) was, on average, close to the AT for all frequencies, demonstrating the effectiveness of masking, as the minimal stimulus intensity difference on the dial was 5 dB and the standard deviation ranged from 2.42 to 3.54 dB.

Tables 3 and 4 show the results for groups G2 e G3.

**Table 3:** AT and MT values (in dBNPS) with TEN at 10 dB better than the AT, obtained in 15 group 2 ears (G2).

Ear	FREQUENCY - HZ															
	250		500		1000		2000		3000		4000		6000		8000	
	AT	MT	AT	MT	AT	MT	AT	MT	AT	MT	AT	MT	AT	MT	AT	MT
1	55		55		65	65	70	70	70	70	70	70	85	85	100	100
2	55		55		65	65	75	75	70	70	80	80	100	100	100	100
3	55		50		50	50	55	55	70	70	85	85	95	95	100	desc
4	70		60		45	45	50	50	75	75	85	85	95	95	90	90
5	40		50		65	65	70	70	75	75	85	85	85	85	95	95
6	55		60		60	60	75	75	70	70	75	75	80	80	95	95
7	75		75		80	80	80	80	80	80	85	85	100	105	100	100
8	50		55		60	60	65	65	60	60	65	65	85	85	75	75
9	45		50		55	55	65	65	70	70	70	70	85	85	90	90
10	70		60		75	75	80	80	65	65	80	80	75	75	85	85
11	80		65		85	85	70	70	60	65	70	70	85	85	90	90
12	50		60		65	65	80	80	85	85	95	95	90	90	85	85
13	30		30		30	30	40	40	50	50	55	55	55	55	60	60
14	30		30		30	30	30	30	65	65	75	75	90	90	75	75
15	60		45		55	55	75	75	85	85	80	80	100	100	90	90

**Legend:** disc auditory discomfort, threshold not assessed AT absolute threshold

**MT** masked threshold with TEN NR no response

**Table 4:** AT and MT values (in dBNPS) with TEN at 10dB better than the AT, obtained in 76 group 3 ears (G3).

Ear	FREQUENCY - HZ															
	250		500		1000		2000		3000		4000		6000		8000	
	AT	MT	AT	MT	AT	MT	AT	MT	AT	MT	AT	MT	AT	MT	AT	MT
1	30		30		30	40	45	55	45	55	60	70	80	90	95	105
2	30		30		30	40	40	50	55	55	60	70	85	95	95	105
3	40		40		35	35	70	80	80	90	85	95	100	disc	90	disc
4	40		40		40	40	60	70	75	85	80	90	105	disc	95	disc
5	45		35		30	30	50	60	85	95	100	Disc	110	disc	NR	—
6	35		35		35	35	75	85	90	disc	105	Disc	110	disc	NR	—
7	40		50		70	80	75	85	75	85	85	95	90	disc	95	disc
8	35		30		40	50	60	70	90	100	90	100	100	disc	NR	—
9	30		30		30	40	80	90	85	95	80	90	90	100	80	90
10	30		30		30	30	80	90	80	90	75	85	85	95	90	100
11	35		35		35	45	80	90	80	90	95	100	115	disc	115	disc
12	40		40		40	50	75	85	80	90	90	100	115	disc	115	disc
13	40		35		85	95	NR		115	NR	115	NR	115	NR	95	NR
14	30		30		75	85	110	NR	NR	NR	NR	—	NR	—	NR	—
15	30		40		55	65	60	70	60	70	75	85	75	85	95	105
16	30		35		50	50	65	75	60	70	75	85	75	85	95	105
17	40		40		70	80	70	80	80	90	95	Disc	85	90	95	disc
18	30		30		50	60	75	85	80	90	100	Disc	90	100	90	100
19	35		40		40	50	75	85	65	75	75	85	85	95	90	100
20	35		40		45	55	70	80	70	80	80	90	100	disc	110	disc
21	30		45		55	65	65	75	65	75	55	65	75	85	90	100
22	30		35		50	60	60	70	60	70	60	70	75	85	95	105
23	35		45		55	55	75	85	85	85	85	95	115	NR	115	NR
24	30		35		50	60	65	75	75	85	75	85	80	90	100	110
25	30		45		55	65	70	80	75	85	75	85	105	disc	95	105
26	30		45		60	70	65	75	65	75	70	80	105	disc	100	disc
27	60		65		65	75	95	105	90	100	NR	—	NR	—	NR	—
28	45		50		65	75	85	95	90	100	100	110	NR	—	NR	—
29	30		40		55	60	75	85	80	90	75	85	95	100	105	disc
30	30		40		55	65	70	80	70	80	70	80	95	100	95	105
31	30		30		30	40	60	70	65	75	65	75	85	95	80	90
32	30		30		30	40	60	70	70	80	80	90	60	65	NR	—
33	35		55		75	85	60	70	85	95	80	90	90	100	NR	—
34	30		55		70	75	75	85	80	90	85	90	90	95	105	115
35	30		45		60	70	80	85	75	80	75	85	110	NR	110	NR
36	35		50		60	65	70	75	70	75	85	90	105	disc	110	NR
37	30		30		35	35	70	80	70	80	80	90	80	90	85	95
38	30		30		35	35	70	80	80	90	80	90	90	100	105	115
39	35		30		50	55	90	95	105	NR	115	NR	110	NR	115	NR
40	30		30		35	45	85	90	95	NR	100	105	110	NR	110	NR
41	35		40		60	65	70	75	65	70	85	95	100	105	105	NR
42	50		55		75	85	110	NR	NR		115	NR	NR	—	NR	—
43	35		50		75	85	75	80	75	80	90	95	95	105	105	115
44	40		35		50	50	55	60	60	70	75	85	90	100	105	NR
45	50		30		30	35	30	35	60	70	70	75	80	90	NR	—
46	35		45		60	65	75	80	90	95	105	NR	110	NR	115	NR
47	30		40		60	65	70	75	75	85	105	115	115	NR	115	NR
48	30		30		60	60	80	85	95	105	105	NR	NR		105	NR
49	30		30		55	65	90	100	95	105	105	NR	100	NR	NR	—
50	30		30		30	30	65	75	80	90	80	90	80	90	90	100
51	30		30		30	30	65	70	85	90	85	95	105	115	105	NR
52	45		45		55	55	90	90	85	85	95	95	90	100	90	90
53	35		40		50	50	70	70	75	85	85	95	95	105	110	NR
54	40		55		65	75	70	80	85	95	95	105	95	105	110	NR
55	35		40		45	45	75	75	75	75	70	75	85	85	90	100
56	35		35		40	50	65	75	70	70	80	80	85	85	100	100
57	35		30		40	40	65	75	70	75	80	80	105	disc	100	disc
58	35		30		35	45	50	50	55	55	60	60	75	75	80	90
59	35		30		30	30	45	45	50	50	70	70	80	90	100	105
60	50		40		30	30	35	35	45	45	60	65	90	95	105	disc
61	30		40		65	65	75	75	70	70	70	70	70	70	65	65
62	30		35		65	65	85	85	70	70	80	80	70	70	70	70
63	35		35		60	60	85	90	90	100	100	105	NR	—	NR	—
64	35		40		60	70	75	85	70	75	80	85	85	90	110	NR
65	45		55		50	60	65	75	65	70	70	75	95	100	100	disc
66	45		45		55	60	75	75	80	90	80	85	115	NR	115	NR
67	35		30		70	80	85	90	85	85	90	100	95	105	110	NR
68	30		30		30	30	50	50	45	55	60	70	100	110	95	105
69	50		45		50	60	40	50	70	80	85	95	110	NR	105	NR
70	30		30		30	30	60	70	55	65	75	85	75	85	110	NR
71	35		40		35	45	50	60	55	65	75	85	100	110	NR	—
72	45		55		70	80	75	85	70	80	75	85	110	NR	115	NR
73	50		45		60	70	65	75	70	80	85	95	95	105	110	NR
74	35		35		60	70	70	80	80	90	85	95	95	105	90	100
75	30		30		65	75	75	85	80	90	90	100	105	disc	115	disc
76	30		30		65	75	70	80	75	85	75	85	115	NR	115	NR

**Legend:** AT absolute threshold MT masked threshold with TEN NR no response disc presence of discomfort, threshold not assessed

According to Moore et al.'s^3^ criteria, we saw no dead regions in all tested ears shown on Table 3. Ears 7 and 11, at 6 kHz and 3 kHz, had MTs 5 dB worse than the AT. Auditory discomfort did not allow investigation at 8 KHz in ear 3. The test for dead regions was considered inconclusive for these three ears.

**Figure 1:**
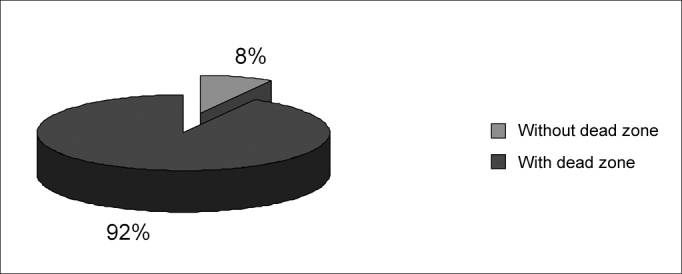
Frequency of occurrence of dead regions in 76 group G3 ears.

**Figure 2:**
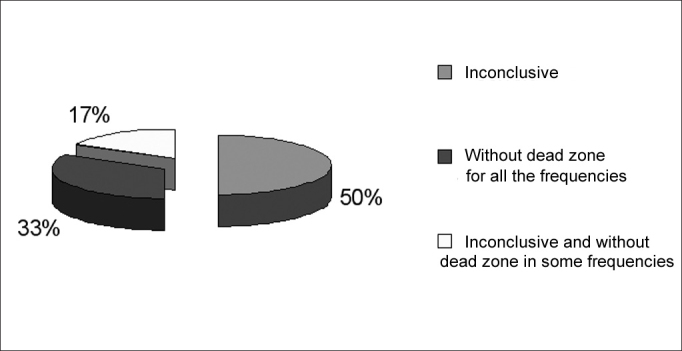
Frequency of occurrence of inconclusive results and with no dead regions in 6 ears with results not indicating dead regions in group G3.

**Figure 3:**
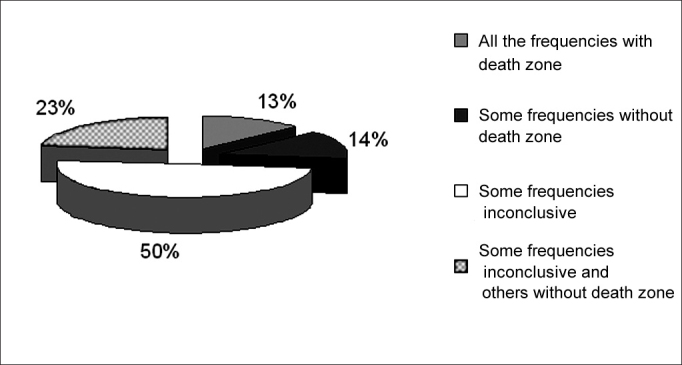
Frequency of occurrence of inconclusive results and with no dead regions in 70 ears with results suggesting dead regions in group G3.

A frequency of occurrence analysis was made in group G3 (Table 4), as shown on Figures 1, 2 and 3.

### Statistical analysis

 

### Comparison of the difference between MTs and ATs by frequency

Statistical analysis using the t test (Ho= A=B and H1= B>A) was applied to group G3, in which the tests suggested dead regions in the cochlea according to Moore et al.'s^3^ criteria. The aim was to check whether dead regions were more frequent at higher frequencies, defined by the difference between the MT and the AT, in cases of sloping sensorineural hearing loss. An added difference between the MT and the AT was assessed at high frequencies. According to our statistical analysis, the difference between the MT and the AT at 8 kHz was not significantly higher than the difference between the MT and the AT at 1 kHz. The same result applied to the difference at 6 kHz and 1 kHz, 8 kHz and 6 kHz, and 4 kHz and 3 kHz. The difference between the MT and the AT was significantly higher at 2 kHz compared to 1 kHz.

### Comparison of the difference between MTs and ATs by frequency

The t test (Ho= A=B e H1= B>A) was applied to group G3 to check whether there was any significant difference between the MT and the AT after applying the TEN test. No significant difference was seen between the MT and the AT at all frequencies.

### Correlation of the difference between MTs and ATs by frequency

Spearman's rank correlation coefficient (rs) (Ho= rs = 0 and H1= rs ≠ 0) was calculated to check whether dead regions could be correlated with different frequencies, in cases of sloping sensorineural hearing loss. Differences between the MT and the AT were reported by frequency in group G3.

The correlation is significant only for differences between the MT and the AT between frequencies of 1 kHz and 2 kHz, 6 kHz and 8 kHz, and 3 kHz and 4 kHz for group G3. The correlation is not significant between frequencies of 1 kHz and 8 kHz, and 1 kHz and 6 kHz.

## DISCUSSION

A 5dB variation over the absolute auditory threshold following presentation of TEN was considered as indicating the absence of dead regions in the cochlea. This variation was observed in the biological calibration procedure for ten normal ears (group G1; Table 2), and in the TEN test on fifteen ears with moderate flat hearing loss (group G2; Table 3) in which this difference was seen on only two ears. Moore et al.^3^ reported a 2 dB to 3 dB variation in the auditory thresholds, standardizing the 5dB difference as an inconclusive result for identifying dead regions. Eguti^8^ reported a 5dB variation on the absolute auditory threshold by presenting white noise during the biological calibration procedure to ten normal listeners.

According to Moore et al.'s^3^ criteria, which also considers a sloping audiometric configuration as evidence of dead regions, we found seventy ears out of seventy-six ears in group G3 with results suggesting dead regions (Figure 1). All of the group G3 audiograms had at least a 50dB difference per octave between two of all tested frequencies. Results showed a significant difference between the MT and the AT for all frequencies.

Statistical analysis showed that the difference between the MT and the AT was higher only for 2 kHz when compared to 1 kHz. Moore et al.^3^ defines a 10 dB difference between the MT and the AT as indicating dead regions in the cochlea. Moore et al.'s papers^3^, ^5^, ^6^, ^13^, ^14^ on dead regions at high frequency in sloping sensorineural hearing loss have showed that generally the estimated dead region limit frequency starts at 2 kHz, which would justify the difference between 2 kHz and 1 kHz we found. Moore^13^ recommended that in sloping hearing loss the TEN test should be applied at 2 kHz and upwards, or in cases where the threshold change would imply in a progression of the degree of hearing loss from mild or moderate to severe.

Equality relations between 8 kHz and 6 kHz, and 4 kHz and 3 kHz could be associated with the closeness of theses frequencies; a larger difference has been observed from 2 kHz upwards, which suggests constancy in the variation of masked thresholds for high frequencies.

Inconclusive results were seen more frequently at frequencies of 6 kHz and 8 kHz (Table 4), mostly due to discomfort, lack of response, or insufficient masking intensity. These results may have influenced the lack of a difference between MT and AT relations for frequencies of 8 kHz and 1 kHz, and 6 kHz and 1 kHz, although Moore et al.^3^ states that unresponsive frequencies for the most intense stimulus level in the audiometer are strongly indicative of dead regions in the cochlea.

Spearman's rank correlation coefficient was calculated for group G3 to check whether dead regions could be associated with different frequencies in sloping sensorineural hearing loss. There was no significant correlation between distant frequencies, such as between 1 kHz and 8 kHz, and 1 kHz and 6 kHz. For close frequencies, such as 1 kHz and 2 kHz, and 3 kHz and 4 kHz for G3, and 6 kHz and 8 kHz, there was a significant correlation, which could be explained by the excitation diffusion phenomenon in the case of dead regions within the nervous fiber response area. According to Henderson et al.,^15^ Bess and Humes,^16^ and Lent,^17^ any frequency and intensity combination represented within this area produces a nervous fiber response. However, when the stimulus is moved upwards or downwards from that frequency, the intensity has to be raised to activate the fiber. In sensorineural hearing loss, where tuning curves are widened, excitation would be distributed more widely close to the threshold in which normal hearing would take place (Evans^18^).

Figure 1 shows that 92% of seventy-six tested ears had results indicating dead regions, which agrees with previous results that suggest a significant prevalence of dead regions in individuals with sloping sensorineural hearing loss.^3^, ^5^, ^6^, ^13^, ^14^, ^9^, ^19^ Summers et al.^19^ used TEN and psychophysical tuning curves (PTC) to assess eighteen ears with moderate to severe sloping sensorineural hearing loss. Results from both tests were similar, showing dead regions in six ears and no dead regions in four ears out of ten ears. Results for the remaining eight ears diverged in one or more frequencies in the TEN test and the PTC, where TEN test results suggested dead regions and PTC did not. The authors explained that the difficulty in listening to noise experienced by individuals with sensorineural hearing loss could be related to the loss of filtering activity due to widened tuning curves, which would raise threshold levels on the TEN test. On PTC, as the noise intensity is very close to the threshold, there would be no difficulty, as the signal-to-noise ratio would be larger. According to Moore et al.^3^ results between PTC and the TEN test were similar in 20 ears with sensorineural hearing loss. Eguti^8^ observed a strong agreement (kappa index of agreement = 96.1%) between Moore's (2001) criteria and masking test results using white noise.

White noise tests for dead regions, however, require further validation, such as comparisons with PTC results. PTC is a proven and internationally accepted method to study cochlear tuning curves (Halpin^20^).

Summers et al.^19^ used a fixed TEN level of 70, 85 or 90 dB/ERB, which for certain thresholds, would be a negative signal-to-noise ratio. We fixed the TEN level at a signal-to-noise ratio of +10 dB, so that there would be no noise filter difficulties.

The TEN test described by Moore et al.^3^ is a simple procedure based on the routine investigation of air conduction pure tone thresholds. However, it does not use a dBNA as an intensity value measure, which is conventionally used clinically in audiogram reports. The TEN value is expressed as dB/ERB, and was calibrated to be equivalent to dBNPS. Therefore, the clinician has to first investigate air conduction pure tone thresholds in dBNA by the audiometer stimulus and then convert it into dBNPS, which requires additional time in the audiological diagnosis routine. An option is to use pure tones in dBNPS recorded on a TEN test CD, as adopted in our study. However, this procedure is feasible only when the aim is to investigate the presence of dead regions, which in itself can only be defined following an audiogram result that shows configuration suggesting dead regions, such as an inter-octave difference equalt to or higher than 50dB.5 Moore^21^ also recommended that the TEN test be applied in individuals that report receiving the pure tone as “noise, and not a whistle,” for users of hearing aids that report no benefits with amplification, to define an indication for short-insertion cochlear implants, and in occupational audiometry as a legal support in cases where high frequencies could be more damaged than suggested by conventional audiometry.

During the TEN test dial readings can by somewhat confusing; according to Moore el al.'s^3^ recommendations, the right channel noise intensity level should be read as 10 dB better than the level indicated on the dial, and that the left channel pure tone should be read as 10 dB worse.

To address these difficulties in the TEN test, Moore^22^ presented a new version published on a CD on the site hearing.psychol.cam.ac.uk - the TEN(HL) - now calibrated in dBNA and with pure tone and noise levels corresponding to dial readings.

Although we saw a significant difference between the MT and the AT at all frequencies, the number of inconclusive results (Figure 1) and the calibration procedure for the TEN test version we used in this study, as well as the results obtained by Summers,^19^ who noted divergences between the TEN test and the PTC for 8 out of 18 ears, led us to question the need for further studies to demonstrate the TEN test sensitivity in detecting dead regions in the cochlea.

As Moore^5^ has suggested, higher TEN thresholds may be due to central problems, rather than dead regions in the cochlea. However, we saw a significant difference between the MT and the AT only for sloping and not for flat hearing losses, which suggests a difference in the detection of pure tones in the presence of noise for individuals with hearing loss at high frequencies and those with flat hearing loss.

## CONCLUSION

A critical analysis of our results in this study allows us to conclude that:

- Dead regions in the cochlea were present in 92% of cases of sloping sensorineural hearing loss and absent in flat hearing loss.
- The TEN test is effective to suggest the presence of dead regions in the cochlea in individuals with sloping sensorineural hearing loss.
